# A Heterojunction Design of Single Layer Hole Tunneling ZnO Passivation Wrapping around TiO_**2**_Nanowires for Superior Photocatalytic Performance

**DOI:** 10.1038/srep30587

**Published:** 2016-07-28

**Authors:** Amir Ghobadi, T. Gamze Ulusoy, Ruslan Garifullin, Mustafa O. Guler, Ali K. Okyay

**Affiliations:** 1 Department of Electrical and Electronics Engineering, Bilkent University, 06800 Ankara, Turkey; 2 UNAM – National Nanotechnology Research Center, Institute of Materials Science and Nanotechnology, Bilkent University, 06800 Ankara, Turkey

## Abstract

Nanostructured hybrid heterojunctions have been studied widely for photocatalytic applications due to their superior optical and structural properties. In this work, the impact of angstrom thick atomic layer deposited (ALD) ZnO shell layer on photocatalytic activity (PCA) of hydrothermal grown single crystalline TiO_2_ nanowires (NWs) is systematically explored. We showed that a single cycle of ALD ZnO layer wrapped around TiO_2_ NWs, considerably boosts the PCA of the heterostructure. Subsequent cycles, however, gradually hinder the photocatalytic activity (PCA) of the TiO_2_ NWs. Various structural, optical, and transient characterizations are employed to scrutinize this unprecedented change. We show that a single atomic layer of ZnO shell not only increases light harvesting capability of the heterostructure via extension of the absorption toward visible wavelengths, but also mitigates recombination probability of carriers through reduction of surface defects density and introduction of proper charge separation along the core-shell interface. Furthermore, the ultrathin ZnO shell layer allows a strong contribution of the core (TiO_2_) valence band holes through tunneling across the ultrathin interface. All mechanisms responsible for this enhanced PCA of heterostructure are elucidated and corresponding models are proposed.

Recently, organic pollutants and their destructive impact on the environment are subjects of an increasing concern in today’s modern society. A tremendous effort has been devoted on developing various technologies to disintegrate toxic organic pollutants^1^,^2^,^3^,^4^. Among all of these approaches, semiconductor based photocatalysis, as an efficient way to convert solar energy into chemical energy without any secondary pollution, has undergone a renaissance since its invention by Fujishima and Honda^5^. In spite of using a wide variety of semiconductors, titanium dioxide (TiO_2_) has been frequently utilized owing to its low cost, nontoxicity, high chemical stability and photocatalytic activity (PCA) under UV irradiation^6^,^7^,^8^,^9^,^10^. To date, several strategies have been utilized to improve photocatalytic/ photoelectrochemical performance of TiO_2_^10^,^11^,^12^,^13^. These approaches can mainly be categorized into: 1) engineering the morphology of the semiconductor catalyst through synthesizing different types of nanostructures such as nanoparticles^13^,^14^,^15^,^16^, nanowires^17^,^18^,^19^,^20^,^21^,^22^, nanosheets^23^,^24^,^25^,^26^, and nanotubes^27^,^28^,^29^,^30^ essentially to enhance both electron transfer capability and surface area, 2) doping with various transition metal cations^31^ (e.g Cu^32^,^33^, Co^34^,^35^, Zn^36^,^37^,^38^,^39^,^40^,^41^, Fe^33^, V^33^, Nb^31^, Cr^2^, etc.), non-metal anions (e.g. H^31^, F^31^,^42^, C^42^, S^42^, and N^42^) and composites with low band gap semiconductors^43^,^44^,^45^,^46^ to extend the absorption edge of light to the visible portion of the spectrum, and 3) introducing novel core-shell nanocomposites in order to extend absorption edge of the core material and reduce the recombination rate of photogenerated electron-hole pairs by selective isolation of the carriers^47^,^48^,^49^,^50^,^51^,^52^,^53^,^54^,^55^,^56^,^57^,^58^,^59^,^60^,^61^,^62^,^63^. In general, a high performance photocatalysis requires not only high surface area to harvest light in a stronger way, but also an efficient architecture prolonging photogenerated carriers lifetime to promote their contribution in photocatalytic process. Among all nano-architectures, the most typical ones that offer high surface area are nanoparticles, nanotubes and nanowires. Although among all of them nanoparticles offer the highest surface area, nanotubes and nanowires show higher PCA mainly due to better charge separation and photocurrent response^64^. It was demonstrated that photoconversion efficiency of TiO_2_ nanowires is higher than that of spherical nanoparticles^65^,^66^.

More specifically, the undesirable charge recombination of photogenerated electrons and holes is often a main limiting factor that lowers the quantum yield of the overall process^1011^. Core-shell structures (semiconductor/semiconductor, semiconductor/metal and metal/semiconductor) with proper band alignment have been regularly employed as the most promising architecture to achieve high PCA. In such a scheme, the selective isolation of charge carriers is achieved through spatial separation of electrons and holes across the core/shell interface in which one type of carriers (often electrons) will diffuse to the shell while its conjugate is confined at the core and therefore their recombination rate is suppressed^44^,^45^,^60^. One of the most recognized (employed) nanocomposite configurations for PCA is TiO_2_-ZnO (or vice versa) core-shell combination^36^,^50^,^51^,^58^,^59^,^60^. In recent years, some researchers have reported TiO_2_ PCA enhancement through doping with Zn^2+^ ions^36^,^41^ or deposition of ZnO^50^,^51^,^58^,^59^,^60^ shell layers. Although, single ZnO layer can also offer good charge transport properties due to its high mobility but it suffers from chemical instability and several bulk and surface defects which lower its overall photocatalytic performance. Therefore, substantial improvement in photocatalytic performance of the pure ZnO (or TiO_2_) can be obtained using core-shell architecture where two metal oxide layers are used to improve chemical and optical properties of the material. Several metal oxides, such as SiO_2_, Al_2_O_3_, or TiO_2_ have been used to make different ZnO nanocomposites. Among all of these metal oxides, TiO_2_ with a wide optical band gap and high chemical stability has energy levels that are matched with that of ZnO. That’s why a core-shell combination of these two materials can offer a high photocatalytic activity through selective charge separation in the core-shell interface. Some of the studies have explained that this enhancement in PCA is attributed to the efficient isolation of electrons and holes. While some others have speculated that the enhancement is related to existence of surface defect states that act as mediators between generated carriers and oxygen containing radicals.

Although the main intention in using shell layer is to reduce recombination rate of carriers, it should be considered that this shell layer often mitigates or completely suppresses the contribution of one type of charge carriers in the overall PCA (mostly the core valance band holes)^44^,^45^. Furthermore, presence of defect states at the core-shell interface or within the bulk of shell layer has detrimental impact on the overall photocatalysis efficiency of the cell. In general, sole retardation of carrier recombination at the core-shell interface is not sufficient to ensure high PCA, but also the charge permissivity of the shell layer should be carefully designed to effectively allow transporting carriers reach the surface, where the reduction and oxidation reactions take place.

Here, we have scrutinized the effectiveness of utilizing ultrathin angstrom thick shell layer on photocatalytic performance of the TiO_2_ nanowire (NW) core. The core-shell heterojunction has been fabricated by combination of hydrothermal growth of single crystalline TiO_2_ NWs core and atomic layer deposition (ALD) of ZnO shell layer. Even though a variety of preparation methods are used to make this shell layer (such as sol-gel^67^, dip-coating^68^, etc), the unique properties of ALD have been exploited to form an ultrathin shell layer. ALD is built on self-limiting sequential surface reactions from at least two gas-phase molecular precursors and this technique offers pinhole-free metal-oxide films with angstrom-scale thickness control. In this work, we demonstrate that conformal coating of ZnO shell layer by merely a couple of ALD cycles (<3 cycles), wrapped around the TiO_2_ core, can intensify the photocatalytic reactions, significantly. It will be shown that the existence of just 1 cycle ZnO shell layer can improve photocatalytic performance of the NW array *via* 1) reducing the recombination rate of the photogenerated carriers by ensuring efficient charge separation at the core-shell interface, 2) improving the collection capability of the structure by reducing the density of oxygen vacancy levels at the interface together with minimizing the transport path for carrier diffusion (owing to ultrathin thickness), and 3) keeping the valence band holes’ contribution up via tunneling across the ultrathin ZnO layer. Different from similar previous studies that are all based on utilization of thick shell layer (~1–10 s of nm)^69^,^70^,^71^,^72^, this work contains several results proving extraordinary performance enhancement in the use of angstrom thick atomic scale shell layer. This paper proposes a new heterostructure design strategy in which employing single ALD cycle of hole tunnelling passivation layer leads to superior photocatalytic performance. The impact of the layer on optical and structural properties of TiO_2_ NWs has been investigated utilizing several characterization methods. In addition to photocatalysis application, the results obtained here are of particular interest to other applications such as photoelectrochemical water splitting and metal-oxide based photovoltaic cells where a substantial increase in the photocurrent can be attained as a result of stronger absorption less recombination and better charge collection capability of the photoanode. This work not only offers a systematic analysis on the effectiveness of this angstrom-thick ZnO layer, but also proposes a new approach for future performance enhanced designs in which the stronger PCA can be achieved while using less material.

## Methods

### Materials

All of the chemicals were used as received without further purification. Titanium (IV) butoxide (Ti(OBu)_4_, 97%, Sigma–Aldrich), hydrochloric acid (HCl, 36%, Sigma–Aldrich) and HPLC-grade water (H_2_O) were used in hydrothermal growth of TiO_2_ NWs on FTO coated glass (7 Ω sq^−1^, Solaronix). Diethylzinc (DEZn, Sigma-Aldrich) and HPLC-grade water (H_2_O) are used as the zinc and oxygen precursors in the ALD process, respectively. The photocatalytic activities of TiO_2_-ZnO heterojunction photocatalysts are investigated by the photo-degradation of methylene blue (MB, Sigma–Aldrich, certified by the Biological Stain Commission) as model organic pollutant in aqueous solutions (40 mL total volume, 20 mM).

### Synthesis of TiO_2_ NW Arrays

TiO_2_ NW arrays are synthesized by a hydrothermal process on FTO glass as described in our previous study^73^. Briefly, FTO glasses are cleaned in sonication baths in ethanol, acetone and de-ionized (DI) water each for 15 min and then dried by N_2_ flow. In a typical process, HCl (20 mL) and DI water (20 mL) are stirred for 10 min in a teflon-lined stainless steel autoclave with a capacity of 45 mL. Then, 0.8 mL of Ti(OBu)_4_ is added to the solution and stirring additional 30 min under ambient conditions. FTO is placed into the autoclave with the conducting side facing up with 45° angle against the wall. After autoclave is maintained at 150 °C for 4 hours, it is allowed to cool at the room temperature. The final product is rinsed extensively with DI water to remove residual solvent, and finally dried in air.

### ZnO Passivation layer of TiO_2_ NWs by Atomic Layer Deposition

ZnO depositions were carried out at 250 °C in ALD reactor (Cambridge Nanotech Savannah S100). Different cycles (1, 2, 3, 5, 10, 20) of ZnO are applied on TiO_2_ NW arrays as an ultrathin layer with the estimated growth rate of 1.3 Å per cycle. Pulse times of the Zn precursor (DEZn at 80 °C) and oxygen source (H_2_O) are both 0.015 s, and kept in the chamber for 10 s.

### Characterization

Scanning electron microscope (SEM, FEI – Quanta 200 FEG) operated at 10 kV and transmission electron microscope (TEM, Tecnai G2-F30, FEI) operated at 200 kV. TEM samples were dispersed in ethanol and prepared on a holey carbon coated copper grid. Selected area electron diffraction (SAED) patterns were used to identify the growth direction and crystallinity of the NWs. TEM-EDX spectra are also recorded for all samples. X-ray diffractometer (XRD) has been carried out by Panalytical X’pert Multi-Purpose and the patterns have been collected in the range of 2*θ* = 20–80° using Bragg–Brentano geometry (Cu K***α*** radiation). X-ray photoelectron spectroscopy (XPS, Thermoscientic K-Alpha, Al K-Alpha radiation, hʋ = 1486.6 eV) has been performed at survey mode by operating flood gun to prevent surface charging with the pass energy and step size set to 30 eV and 0.1 eV, respectively. Photoluminescence (PL) measurements have been performed using Cary Eclipse Fluorescence Spectrophotometer with an excitation wavelength of 300 nm, and the emission spectra were recorded between 360 nm and 650 nm. For optical characterization of the NWs, UV-Vis-NIR spectrophotometer (Cary 5000, Varian) was employed.

### Photocatalytic Activity (PCA) testing

The PCA of the TiO_2_ NWs with varying ZnO shell thickness was measured by the degradation of MB aqueous solutions and compared with the samples without ZnO passivation layer. The samples (1.5 mg each) were removed from FTO samples using razor blade, then dispersed in 2500 μL of 68.4 μM MB solution in quartz cuvettes and exposed to UV irradiation (8 W, UVLMS-38 EL, 365 nm) from a distance of c.a ~10 cm. During the experiments, composites were at the bottom of the cuvette, and hence did not interfere with the data acquisition. The change in the absorption peak of MB (365 nm) as a function of exposure time (t) to UV light was investigated.

## Results and Discussion

**Figure 1:**
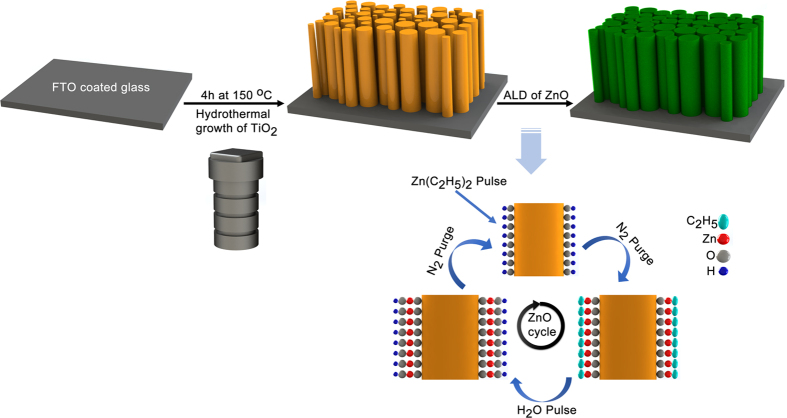
Schematic illustration for the formation of TiO_2_/ZnO heterostructure through a two-step process on FTO coated glass substrate: hydrothermal growth for TiO_2_ NWs core, followed by atomic layer deposition of ZnO shell.

**Figure 2:**
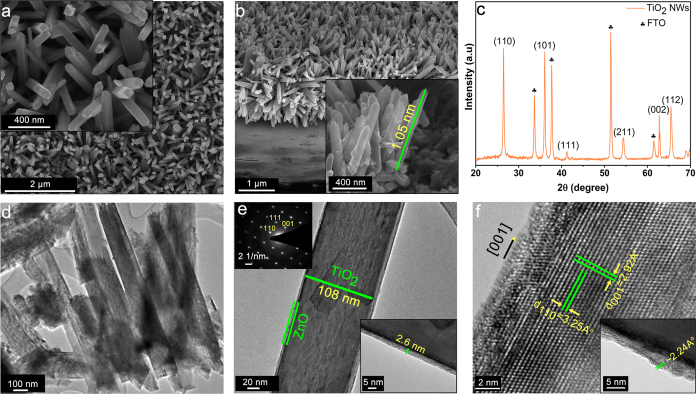
ZnO (20 ALD cycles)-coated rutile TiO_2_ NW arrays (**a,b**) SEM images (top-view and side-view). The inset shows magnified images. (**c**) XRD pattern with the prominent (110) rutile peak of TiO_2_ NWs. (**d**) TEM and (**e**) HRTEM image of an individual ZnO coated NW with high magnification interface, green contrasts corresponding to TiO_2_ core and ZnO shell, respectively indicating the existence of a 2.6nm-thick ZnO shell layer. (SAED pattern and tip angle in the insets). (**f**) HRTEM images proving the lattice fringes, crystalline direction, formation of single crystalline TiO_2_ and polycrystalline ZnO.

**Figure 3:**
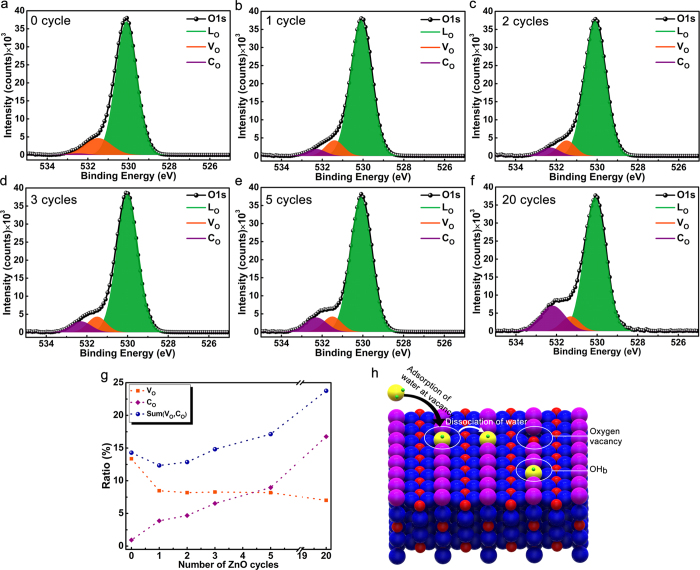
(**a–f**) Deconvoluted O1s XPS core level spectra of all TiO_2_-ZnO core-shell samples fitted with three components, at 530.0, 531.4, 532.4 eV for lattice oxygen (L_O_), oxygen vacancy or defect (V_O_) and chemisorbed oxygen (C_O_), respectively. (**g**) The percentage of areas under the deconvoluted peaks (L_O_, V_O_ and C_O_) to the total area of O1s as a function of ZnO ALD cycles. (**h**) Representative image of adsorption and dissociation process of water molecules and their relation with surface defect states.

Figure 1 (drawn in SOLIDWORKS^®^ software) is a schematic describing the preparation route for TiO_2_-ZnO photocatalyst *via* one-step hydrothermal synthesis of TiO_2_ NW core followed by atomic layer deposition (ALD) of ZnO shell. Scanning electron microscopy (SEM) and transmission electron microscopy (TEM) have been employed to characterize the morphology and dimensions of the structures. SEM images, top and cross sectional views (Fig. 2a,b), demonstrate densely packed NWs with lengths of 0.9–1.6 μm and widths of 80–150 nm. Non-destructive analysis technique of X-ray diffraction (XRD) has been utilized to further investigate the crystallinity phase of the photocatalyst semiconductor. XRD pattern of TiO_2_-ZnO structure exhibits the rutile crystalline phase for TiO_2_ where positions of the peaks and their miller indices have been annotated briefly in the Fig. 2c and the prominent peak of (110) indicate the most preferential crystal plane for the TiO_2_ NWs array growth. It should be noted that there is no trace here which belongs to ZnO since there is no measurable signal from this ultrathin layer in our experimental configuration. Additionally, selected area electron diffraction (SAED) data are also collected to confirm the growth direction and crystallinity of the NWs. High-resolution TEM (HRTEM) images (Fig. 2e,f), taken from one of the NWs presented in Fig. 2d, illustrates that TiO_2_ NWs have single preferred orientation, and ZnO is grainy with random crystalline directions. Therefore, grown TiO_2_ NWs are single crystalline and ZnO shell layer is polycrystalline. The measured thickness of ZnO layer is ~2.6 nm for 20 ALD cycles which is consistent with our previous study^73^. In Fig. 2f, HRTEM image further reveals that the TiO_2_ NWs grow along the [001] direction with lattice fringes corresponding to a d–spacing of ~3.25 and ~2.92 Å for the (110) and (001) facet crystals, respectively. In addition, the polycrystalline ZnO has a c-axis lattice spacing of ~2.24 Å (Figure S1, Supporting Information). These values match the literature and support the formation of tetragonal rutile phase of TiO_2_ and wurtzite type hexagonal ZnO^74^. Moreover, energy-dispersive X-ray spectroscopy (EDS) analysis is performed in scanning TEM (STEM) mode (Figure S2), representing the main compositions of the core-shell heterostructure which are zinc, titanium and oxygen constituents. The oxygen peak and carbon peak, which is unavoidable carbon contamination, are detected in the low energy part of the spectrum and the copper signal originates from TEM grid. Investigations on constituent elements at the surface of the samples and presence of the surface defects at the core-shell interface have been performed using X-ray photoelectron spectroscopy (XPS). The existence of ultrathin ZnO layer on the TiO_2_ NWs has been confirmed by collecting Ti2p and Zn2p spectra at the core-shell interface. As it can be seen from Figure S3, the intensity of the Zn2p peaks is intensified moving to thicker layers. In order to investigate the density and nature of the surface defects in the TiO_2_-ZnO heterostructure, XPS spectra (Fig. 3a–f) for O1s are also recorded and fitted with three Gaussian curves centered at the binding energies of 530.0, 531.4, 532.4 eV for lattice oxygen (L_O_), oxygen vacancy or defect (V_O_) and chemisorbed oxygen species (C_O_), respectively^73^. The low binding energy peak (L_O_) is from the lattice oxygen atoms (O^−2^) in a fully-coordinated TiO_2_ with the Ti^+4^ ions mainly in the bulk. The medium binding energy peak (V_O_) is attributed to the oxygen (O^−^, O^−2^) vacancy in the matrix of the metal-oxide, and high binding energy peak (C_O_), is the loosely adsorbed, dissociated oxygen or OH species which is from O_2_, H_2_O or M-OH on the surface of TiO_2_. XPS analysis indicates that just one cycle deposition of ZnO on TiO_2_ NW surface substantially reduces the V_O_ peak and a small shoulder of C_O_ is identified. As the ZnO shell gets thicker, C_O_ continues to increase, but V_O_ stays almost constant (as shown in Fig. 3g). On the other side, when V_O_ states are formed on the surface of TiO_2_ (110), it is likely that two excess electrons associated with each oxygen vacancy are transferred to the empty 3d orbitals of the neighboring Ti_5c_ ions, forming two Ti^3+^ sites. In the other words, the existence of oxygen vacancy gives rise to two excess electrons. Although in principle these excess electrons can be localized at any Ti atom, they are believed to preferentially occupy specific Ti–3d orbitals, thus formally creating Ti^3+^ sites^31^,^75^,^76^. On the other word, when a crystal of Ti^4+^O2^2−^ loses one oxygen atom, the two electrons of the O^2−^ ion change two Ti^4+^ to two Ti^3+^ ions^77^.

However, there is no sign attributed to this Ti interstitials in the Ti2p spectrum. Therefore, the dominant type of surface defects is V_O_s. On the other hand, water is known to fill V_O_ states for temperatures above 187 K^78^ and form two hydroxyl groups (OH_b_) bound to two neighboring surface oxygen atoms^79^. Considering the ALD process, the substrate is initially exposed to gaseous precursor molecules of water vapor. Based on the calculation of free energy by classical nucleation theory, most of the water derived hydroxyl groups such as OH radicals and H_2_O will be chemisorbed near imperfections such as defects (like V_O_ levels) and grain boundaries^80^. As schematically illustrated in Fig. 3h, water molecules will be first chemisorbed at oxygen vacancies and then subsequent H ion will move to the neighboring oxygen. Therefore, the first exposure of water vapor to the TiO_2_ surface is responsible for passivation of surface vacancies and subsequent cycles just add detrimental effects by introduction of chemisorbed oxygen radicals acting as trap states across the band gap of TiO_2_. To have a comprehensive comparison, the relative areas for each of L_O_, V_O_ and C_O_ have been determined and plotted in Fig. 3g. After introduction of ZnO shell, the ratio of V_O_ has considerably dropped but stayed constant for subsequent cycles. However, the density of C_O_ has linearly risen up by adding the ZnO cycles. The sum of V_O_ and C_O_ has its minimum amount for 1 cycle coated sample and it goes to higher levels than bare sample starting from 3 cycles. Therefore, it can be speculated that just first 2 cycles are able to efficiently passivate surface traps where the advantages of ZnO shell layer outweigh its drawbacks.

**Figure 4:**
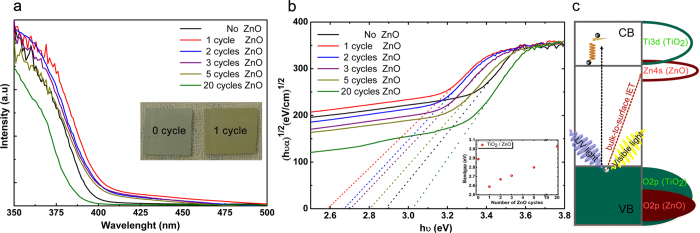
(**a**) Absorption spectra of the bare and ZnO coated samples. Inset shows yellowish color for 1 cycle coated sample, (**b**) Kubelka-Munk-transformed diffuse reflectance spectra of the different ALD ZnO cycles coated TiO_2_ NW arrays used for the estimation of the optical band gap and corresponding estimated band gaps (inset), (**c**) an illustrative representation for the involved absorption mechanisms.

To explore the impact of different number of ZnO ALD cycles on the optical properties of TiO_2_ NWs array, the change in the absorption and optical band gap of the samples upon coating with shell layer has been explored. Considering the thick TiO_2_ NW core and ultrathin ZnO shell, it is expected that all samples should have strong inherent absorption spectra at the UV range associated with electron transition from valence band (VB) to the conduction band (CB) of TiO_2_ (interaband transition, O2p → Ti3d). Also, a weak (and in some cases broad) absorption at the visible spectrum can be observed, which is mainly due to the sub-band transitions mediated by surface defect states^33^,^36^,^81^,^82^,^83^,^84^,^85^. However, the effect of the semiconducting shell layer on the absorption of TiO_2_ is disclosed mostly at the shoulder (around 400 nm) where the existence of this shell layer can provide an extension in the absorption edge of the heterostructure^32^,^33^,^35^,^36^,^86^. In order to provide better qualitative comparison, using transmission data obtained from UV-VIS spectrometer, the absorption spectra and optical band gaps of the samples have been experimentally determined via extrapolating the linear portion of Kubelka-Munk function, (α*hϑ*)^1/2^ versus photon energy, (*hϑ*), as shown in Fig. 4a,b, where *α* is absorption coefficient. The inset in Fig. 4b presents estimated optical band gap (*E*_*g*_) values calculated from the above expression. Although the optical band gap of the bare rutile TiO_2_ NW arrays is found to be 2.89 eV, that for ZnO coated samples exhibit a red-shift where just 1 cycle induces a reduction in energy band gap by 11.58% to 2.59. However, for thicker shell layers a gradual increase in the effective optical band gap is recorded up to 20 cycles, where it reaches a value of 3.03 eV which is even higher than that of bare TiO_2_. Some of the previous reports proposed the band gap narrowing of TiO_2_-ZnO core-shell heterostructure^36^ while others report the opposite, regardless of the amount of doping. Similar to our case, several studies have also reported a change for the optical band gap of the TiO_2_ upon ZnO (or Zn^+2^) deposition in that for low Zn^+2^ contents band gap reduces and then it widens at higher content^37^,^51^. The reduction in the band gap of the first ALD cycle can be attributed to quantum confinement effect in the ultrathin ZnO shell (Fig. 4c). In previous study^73^, we found that a type II band alignment exists at the core-shell interface in which the conduction band of ZnO is positioned at an energy level 0.3 eV lower than that of TiO_2_. Therefore, the formation of Zn4s localized band states just below the CBM of TiO_2_ results in an exitonic transition between TiO_2_ VBM (O2p) and ZnO CBM (mainly Zn4s)^82^. On the other hand, it can be seen that the absorption in visible region (both near bandgap region and the low energy region (<3 eV)) is also increased by 1 cycle ZnO deposition. To address this unprecedented change, we first need to look at the XPS results where the findings show a partial passivation of surface defects upon deposition of ZnO layer. Therefore, the enhancement in visible response of coated samples can be explained by considering both absorption mechanisms; 1) TiO_2_ surface defect states mediated and 2) ZnO CB mediated absorption. In the case of 1 cycle coated ZnO shell layer, this exitonic transition has highest probability of occurrence. Therefore, together with extension of UV absorption edge toward higher wavelengths, these localized states can also contribute in the absorption of the visible light. Previous reports demonstrate that surface modification by ultra-small metal oxide nanoclusters (such as NiO, FeO_x,_ (TiO_2_)_n_, Cu_2_O, Ga_2_O_3_) endows TiO_2_ with visible spectrum activity^31^,^52^,^53^,^54^,^56^,^87^,^88^,^89^,^90^. It has been found that reduction in the band gap of the NiO nanoclusters modified TiO_2_ stems from the rise in the top of the valence band^56^,^88^. The same reasoning was employed to elucidate band gap narrowing of FeO_x_-modified TiO_2_^90^. Similarly, DFT simulations reveal that deposition of Cr_2_O_3_^89^, Cu_2_O^52^, TiO_2_^53^, Ga_2_O_3_^54^, and Mo_2_O_4_^89^ can cause reduction on the effective *E*_*g*_ mainly due to the formation of localized impurity energy states within the band gap. Moreover, in some papers, it is speculated that this unprecedented change can be associated to the advent of oxygen defect states (color centers)^42^,^81^,^82^,^85^,^91^,^92^. Although the mechanistic details of TiO_2_ band gap narrowing (or widening) is not fully understood yet, it generally depends on different factors such as nature and concentration of the dopant impurities, crystalline phase of the TiO_2_, and energy band alignment across the materials interface. This can also be confirmed by comparison between the colors of 1 cycle coated and uncoated samples depicted at inset of Fig. 4a. The yellow color for the coated sample is another evidence for the extension of absorption edge toward visible region. On the other side, the slight increase in the effective optical band gap for consecutive ZnO cycles can be explained by Burstein-Moss effect^93^. Based on this effect, increase in the electron concentration beyond a threshold leads to partial filling of conduction band states. In our case, therefore, some of the lowest conduction band states of TiO_2_ will be occupied with these dopants localized states and conduction band edge will be shifted to higher energies (leading to higher effective band gaps)^37^,^51^. This result is of particular interest to photoelectrochemical water splitting where stronger absorption of TiO_2_ is highly desired. Fundamentally a high performance photoelectrochemical cell should fulfill the requirements of strong absorption, low recombination, high chemical stability and high catalytic activity. Among all types of semiconductors, metal-oxides are the most typically employed ones for water splitting, mainly due to their chemical stability and low recombination rates. However, they suffer from inherent weak absorption which includes just the UV portion solar spectrum. Our strategy using ZnO shell layer is able to improve this deficiency and by this way high performance photoelectrochemical cell can be attained. The photocatalytic activity of the metal oxide semiconductors depends on various factors such as surface area, crystallinity, composition, lattice defects (bulk and surface) and surface adsorbents or bound complexes^94^. Among all, the surface properties of the semiconductor layer are of particular importance in conjunction with the photocatalytic reaction kinetics, because this process mostly takes place on the semiconductor surface. For the heterostructure architecture, the existence of the surface defects at the interface, with an energetic location within the band gap of the semiconductor, provides a pathway for carriers’ trapping/recombination and eliminates their participation in photocatalytic reaction. In principle there are four main types of defects on TiO_2_ which can be classified as: oxygen vacancies (V_O_’s), titanium vacancies (V_Ti_’s) (surface defects), interstitials (Ti_i_ and O_i_), and antisites which are mostly formed in the bulk of material^31^,^53^,^55^,^83^,^92^,^95^. There are several reports which propose Ti_i_’s as the active sites of TiO_2_ surface^81^,^84^,^96^, while the others give this functionality to the V_O_’s^7^,^60^,^97^,^98^. The fact that which of these defects is dominant depends on the preparation method of the material; under Ti reach conditions V_O_’s are the most favorable defects to form^60^,^81^,^99^. Therefore, it is expected that introduction of the TiO_2_ in the solution, during the hydrothermal growth, makes V_O_’s the dominant defect state in the crystal lattice formation. This conclusion has been already confirmed by XPS measurement in which the O 1s spectra showed the existence of V_O_’s on the as prepared samples, while there was no sign of Ti_i_ in Ti 2p spectra.

**Figure 5:**
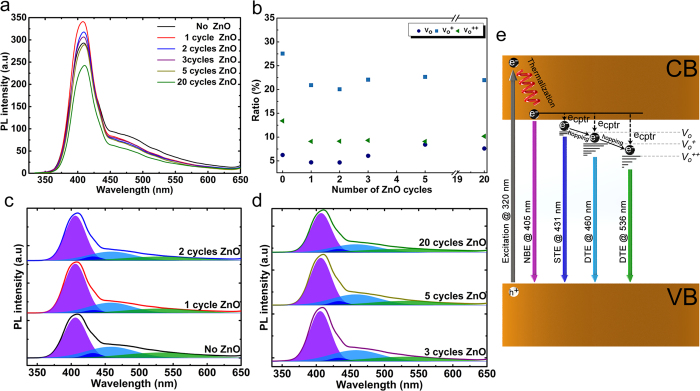
(**a**) PL spectra for the coated and bare samples at an excitation wavelength of 320 nm, (**b**) the ratio of peak intensities (I_STE_/I_NBE_ and I_DTE_/I_NBE_) for the (**c,d**) corresponded deconvoluted peaks and (**e**) depiction of the processes associated with each of these peaks.

In this context, photoluminescence (PL) spectroscopy is a useful technique to disclose the density of surface defects on TiO_2_ surface and the effect of ZnO shell on the passivation of these states^94^,^100^. PL spectra of all samples, obtained at an excitation λ of 320 nm at room temperature, have been shown in Fig. 5a. As it can be clearly seen, PL spectra for all the cases exhibit the same spectral shape showing that the emission from the heterostructure is mainly due to the TiO_2_ defects. Considering the ultrathin thickness of ZnO layer and self-terminating growth of ALD, it is expected that not only ZnO layer does not induce new defects states^101^ but it also passivates the oxygen vacancies on TiO_2_ surface quenching their emission. In this regard, the PL spectra for all samples reveal a narrow strong emission in the UV region and a broad but relatively weak response in visible regime. The UV emission peak, centered at around 405 nm, originates from the interband transition of electrons across the conduction band minimum (CBM) and valence band maximum (VBM) (near band edge emission (NBE)). On the other hand, the broad visible emission stems from two sources; the low energy part is due to shallow trap state emission (STE) and the high-energy emission originates from deep trap states (DTE). As it can be seen the UV related peak reaches its highest peak for 1 cycle coated ZnO sample with a considerable jump compared to uncoated one. This peak represents a decreasing trend in its intensity for the additional cycles. On the other hand, the broad visible emission and its attributed peaks drop abruptly with deposition of first ZnO cycle and reduce gradually moving to higher cycles. From XPS data, we found that ALD ZnO layer can effectively passivate surface traps (Fig. 3). Therefore it is anticipated that visible emission of the coated samples should be quenched as an outcome of this passivation. In other respects, the existence of chemisorbed oxygen radicals, which have linear upward trend as a function of ZnO cycles, can lower the amount of NBE intensity via introduction of non-radiative recombination centers across the TiO_2_ band gap. For an in-depth understanding of position and density of each trap state, the PL spectra of the samples have been deconvoluted to 4 peaks by Gaussian fitting^32^,^81^,^102^. The UV emission peak at 405 nm models NBE peak, and STE peak at 431 nm is associated with a donor type oxygen vacancy located below the CBM^32^,^102^. The other peaks at 460 nm and 536 nm are ascribed to color centers, called F centers (V_O_^+^ and V_O_^++^). These color centers, which are mainly deep trap states, are different types of charged trap states with one and two electrons trapped in their oxygen vacancies, (respectively V_O_^+^ and V_O_^++^)^32^,^102^. It should be pointed out that there is no peak assigned for Ti_i_, considering the XPS results. According to the results of this fitting, singly ionized surface oxygen defect states (V_O_^+^) are the main cause of visible emission. To get a better qualitative comparison the ratios of STE/NBE and DTE/NBE have been illustrated in Fig. 5b,c. Upon deposition of the ZnO shell, these ratios have declined drastically. Therefore, similar to previous results, these findings are also in line with the fact that 1 cycle ZnO shell represents a passivation capability. This is also a superior result for photoelectrochemical water splitting and metal-oxide based solar cells where a substantial improvement in the photocurrent can be achieved due to reduction in the carriers’ recombination/trapping and enhancement in the collection efficiency of the cell.

**Figure 6:**
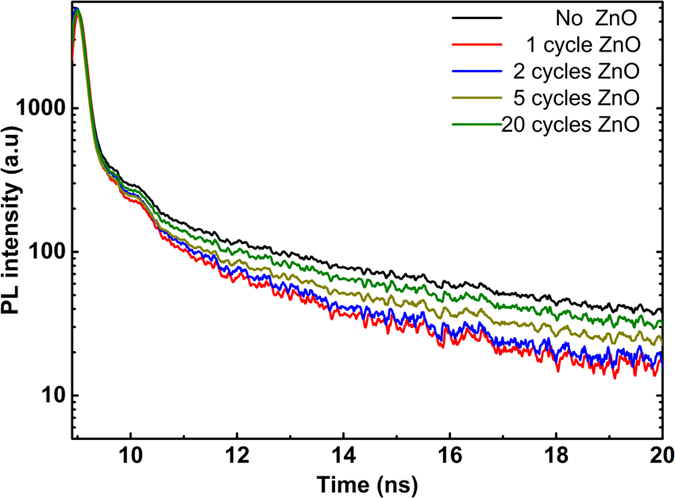
TRPL spectra for different ALD ZnO cycles coated samples representing the surface passivation and charge separation capability of the heterostructure.

Another major factor, affecting the efficiency of photocatalysis is carrier separation capability of the design. In particular, the introduction of secondary semiconductor as the shell at heterostructure architecture is a promising strategy to spatially separate electrons and holes in order to mitigate their recombination. This junction, with proper band offsets, can provide an efficient charge separation, mimicking the pathway in natural photocatalysis. To gain better insight into performance of our core-shell structure, time resolved PL (TRPL) study has been carried out. As it can be noticed from Fig. 6, wrapping TiO_2_ with the first cycle of ALD-coated ZnO will fasten excited state carriers decay and additional cycles will prolong the photoexcited carriers’ photoluminescence decay lifetime. As expected, the decays for all coated samples show faster features which are a result of surface defects passivation of TiO_2_. On the other side, the increase in the lifetime of consequent ZnO cycles can be explained considering the band alignment of the semiconductors at the heterojunction interface. Based on our previous report^73^, TiO_2_-ZnO core-shell structure has type-II band alignment. Thus the excited electrons will diffuse into the conduction band of ZnO while their conjugate (holes) will be blocked at the TiO_2_ valance band. This separation is responsible for extending electron lifetimes at the coated samples. These introduced delay rates are also in line with PL results in which adding subsequent cycles has reduced the NBE emission.

**Figure 7:**
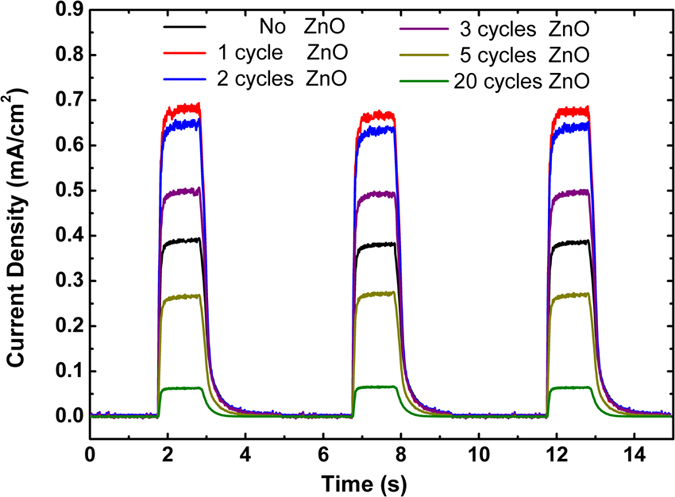
Photocurrent response of the bare and different cycles of ZnO coated TiO_2_ samples.

In a single semiconductor the probability of carriers recombination due to lack of proper separation of electrons and holes is a paramount limiting factor in the overall photocatalytic process. An effective spatial carrier separation can be obtained by a carefully designed core-shell heterostructure. In this regard, the efficiency of a PCA strongly depends on factors such as the band gap of semiconductor catalysts and their corresponding band offsets, crystallinity of each layer and the most importantly, the functionality of the interface where the existence of undesired recombination sites (surface defect states) can hamper carriers transport toward the shell surface to participate in photocatalysis. Therefore, understanding charge transport properties of these layers is of great importance. To investigate this property, the photocurrents of all samples are measured at a zero bias that is equivalent to photocatalytic conditions. For this, we monitored the transient photocurrent as a function of time upon switching off the light. As the light source, we used photon flux of the standard air mass (AM) 1.5G solar spectrum and the test device is a TiO_2_/ZnO core-shell photoanode which is sandwiched with a platinum counter electrode using a cell holder. The internal space of device is filled with 1 ml syringe electrolyte through the backfilling technique. The photocurrent value rises up abruptly from dark values upon switching the light on, as shown in Fig. 7. As it can be seen, the photocurrent amounts for 1 cycle coated sample is the highest and this amount decreases gradually moving toward higher cycles. This result is in line with previous findings where an efficient reduction in the interface trap states guarantees the collection of the photogenerated carriers at the surface. Hence, this measurement is also showing that although first ALD cycles can efficiently improve charge transport properties of the layer but substantial cycles would hamper this property.

Having scrutinized the impact of ultrathin ZnO shell layer on the material and optical properties of TiO_2_ NWs, the involvement of these finding in the PCA of the heterogeneous structure is the next step to get more enlightenment on the physics behind this reaction. We have therefore evaluated PCA of the TiO_2_ NW arrays in the presence of ZnO shell layer, carried out by degradation of methylene blue (MB) dye.

**Figure 8:**
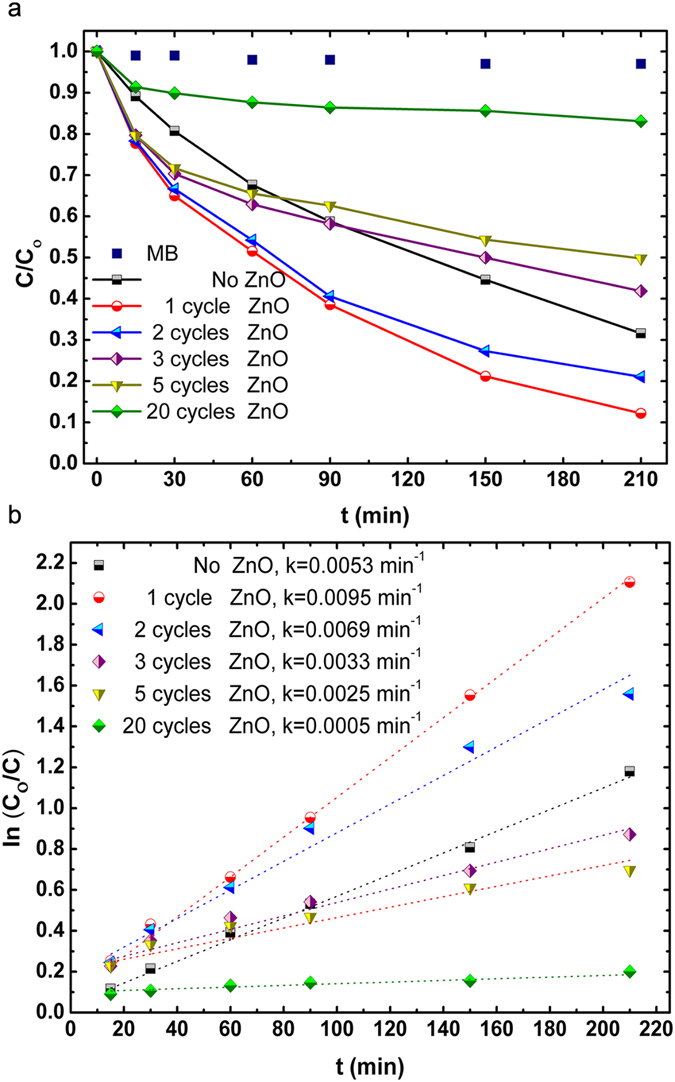
(**a**) PCA of MB aqueous solution in the presence of different ZnO cycles and no catalyst cases (co = 68.4 μM) as a function of the irradiation time. (**b**) Kinetic plots for TiO_2_- ZnO core-shell NW arrays with varying shell thicknesses and comparison in MB degradation rate constants of photocatalysts. The photocatalytic activities of TiO_2_-ZnO composite structures with different ALD shell cycles are evaluated by degradation of MB aqueous solution under a UV-light irradiation. (λ = 365 nm).

Figure 8a shows photocatalytic degradation profile of MB for all samples, the plot includes a normalized concentration (C_O_/C) replaced with a normalized absorbance (A_O_/A). It can be observed that direct photocatalysis of MB, for the blank testing, is negligible under UV illumination, which points out that MB solution was stable during the experiment. As it can be demonstrated from the Figure, the sample with 1 cycle ALD ZnO layer shows superior PCA as expected. In the case of 2 cycles the graph shows small decline in PCA compared to 1 cycle and afterward, there is a noticeable drop at 3 cycles where degradation rate is lower than that of the bare TiO_2_. For the samples, thicker than 3 cycles, the photocatalysis capability falls back exponentially and reaches its lowest level at 20 cycles. To provide a better qualitative comparison between samples, degradation rates and decay times of MB have been extracted and plotted. Figure 8b illustrates the variation of ln(C_O_/C) curves with degradation time for all samples which is a linear relation as expressed by Langmuir-Hinshelwood model (L-H model) and follows pseudo-first-order kinetics with the kinetic reaction expressed as A_t_=A_O_·e^−kt^ or ln (C_O_/C)=kt, where k (min^−1^) is the degradation rate constant (or pseudo-first-order rate constant), C_O_ and C are initial dye concentration and dye concentration at a time (t), respectively. The decay time for 1 cycle coated sample is k=0.0095 min^−1^ (105.26 minutes) which is approximately 20 times faster than 20 cycle coated sample (k=0.0005 min^−1^).

The same experiment is also repeated for the visible light driven photocatalysis, as shown in Figure S4. As expected 1 cycle coated sample has the highest MB degradation rate while this capability declines for thicker ZnO layer where almost no degradation is recorded for 20 cycles thick layer. Similarly, bare TiO_2_ depicts very poor MB degradation capacity due to its weak visible light absorption.

For get deeper insight into these results, It is essential to rewrite the basic photocatalysis mechanism firstly introduced by Izumi^103^. The photocatalytic process is initiated with generation of an electron in CB or a hole in the VB upon illumination with the photons having an energy equal to or greater than the band gap of semiconductor photocatalysts^48^,^104^. Following the formation, electrons and holes can easily be transferred to electron and hole acceptors simultaneously, provided that the respective energetic requirements are fulfilled. Throughout the multiple steps, these photoinduced carriers facilitate the formation of hydroxyl radicals and these highly reactive species eventually oxidize the organic pollutant. The proposed possible photocatalytic degradation steps of these adsorbed molecules in MB aqueous solutions using TiO_2_-ZnO core-shell composites can be described in eqns (1)-(13)^6^,^105^,^106^,^107^. This process consists of complex sequence of reactions including several interface transfer processes^108^. Here, photogenerated electrons, accumulated on the surface of ZnO, primarily react with adsorbed oxygen molecules (dissolved in water) to produce highly oxidative superoxide radical anions (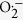)). Further, this molecule is easily protonated by H^+^, which is product of water ionization, and forms hydroperoxyl radicals HOO., and finally H_2_O_2_. Holes at the VB, however, reacts with surface hydroxyls or adsorbed water and generate strong oxidizing agent HO., hydroxyl radical, to eventually oxidize the organic pollutant. The overall photocatalytic efficiency strongly depends on the highly active oxidation species (HO. and 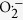) which react with the organic pollutants and the final products are mainly carbon dioxide, water and inorganic ions.


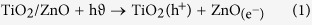



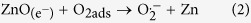



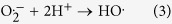



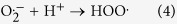



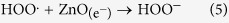



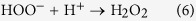



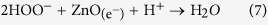



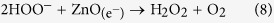



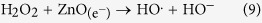



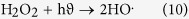



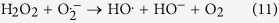



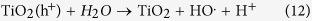



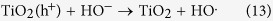


**Figure 9:**
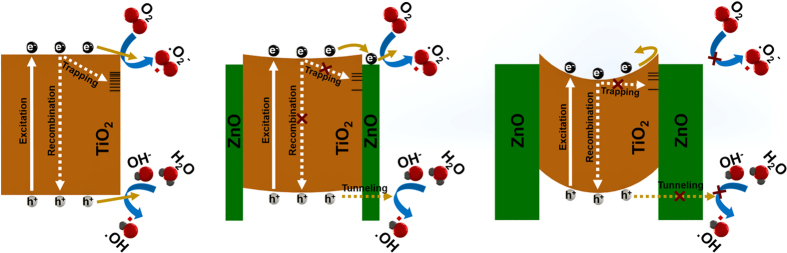
The processes associated with PCA of no, thin and thick ZnO shell layers coated TiO_2_ NWs. The impact of ZnO thickness in different optical and electrical mechanisms at the interface of the heterostructure.

To elucidate the mechanism responsible for this imperative change on the TiO_2_ NW photocatalytic reaction upon deposition of ultrathin ZnO layer, recyclability of the prepared samples have been evaluated for 5 runs since it has been shown in previous studies that PCA of ZnO can be considerably mitigated due to photo-corossion at the surface^109^. According to the Figure S5, bare TiO_2_ represents the most recyclable performance keeping almost same degradation efficiency after 5 recycles. On the other side, ZnO coated samples are experiencing a gradual decrease in degradation capability of MB dye and this reduction is more pronounced for thicker layers where the surface is entirely covered with ZnO shell layer. However, this process does not show a severe photo-corrosion in the ZnO shell layer. In the next step, to understand this unprecedented change, the band diagrams and possible reactions for bare and heterojunction samples have been provided in Fig. 9. Looking back to XPS and PL results, the introduction of initial ZnO cycles (1 and 2) will reduce density of surface traps in TiO_2_. Consequently, the probability of the electron trapping/recombination, while passing across the interface, reduces. It is worthy to mention that several reports have claimed the positive contribution of these surface defects on the photocatalysis reaction^7^,^110^. Based on the findings of these studies, the surface trap states with suitable energetic locations across the band gap of the TiO_2_, can mediate the electron reaction with oxygen-containing molecules. However, this is no longer valid for our case in which electrons are trapped at the interface and they do not have the chance to capture these oxygen species on account of the physical isolation of NWs with ZnO shell layer. So, they can just add detrimental impact on the system and reduction of their density is desired for our architecture. Moreover, the ultrathin shell layer with proper band offset ensures an efficient charge separation while minimizing electron diffusion lengths^45^,^111^. Together with electrons, the influence of holes in the overall PCA should also be taken into consideration. For a typical heterojunction structure, the charge separation is achieved by blocking one of the carriers at the core but letting its conjugate to diffuse into shell layer. Thus, through this isolation, contribution of one of the carriers in the PCA is suppressed^44^,^45^,^112^. However, in our case, holes can tunnel through this angstrom-thick shell layer and participate in the photocatalysis. It has been already demonstrated^113^,^114^ that first ALD cycle serves for passivation of sub-band-edge surface states and afterwards subsequent cycles result in an exponential fall off in electron tunneling probability with increasing barrier layer thickness.

Although the first ZnO ALD cycles add superior optical properties to the NWs and significantly strengthen the PCA of TiO_2_, as the shell layer thickens, an exponential fall off in the PCA is observed. This phenomenon can be attributed to various reasons. The first, as it has been explained in Fig. 3, thicker ZnO layers have more chemisorbed oxygens at their grain boundaries. These oxygen species act as a new recombination centers and trap electrons before reaching to the reaction surface. The second, according to previous reports, oxygen containing species tend to be chemisorbed on grain boundaries of the host surface via free electron captures^115^,^116^,^117^. Consequently, these adsorbed negative oxygen radicals induce a positive potential on the host material and deplete the surface electron states by reducing the density of the free carriers in the vicinity of the surface. This, by itself, facilitates the formation of space charge region and causes surface band bending at the heterojunction interface. Thus, considering the fact that density of chemisorbed oxygen rises up with thickening of ZnO shell layer, it is speculated that surface band bending, caused by chemisorbed oxygen, will hinder electron diffusion to the shell surface. In addition to all of these effects, thicker shell layers reduce the probability of the hole tunneling and quench its contribution to the photocatalytic reaction^45^,^113^. These are all contributing reasons for poor performance of thick ZnO coated samples.

**Figure 10:**
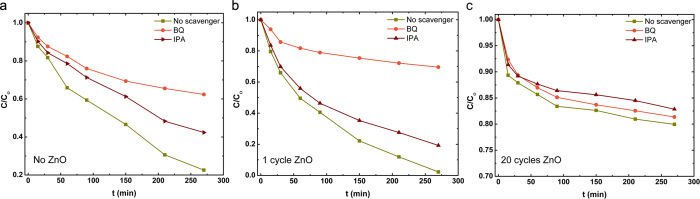
Effect of radical scavenger on the degradation of MB (IPA for ˙OH and BQ for O2˙^−^, respectively) for (**a**) bare, (**b**) 1 cycle coated ZnO and (**c**) 20 cycle coated samples.

To evaluate this hypothesis and to understand which of active species has the main role in MB degradation, photoactivity experiments under UV irradiation in the presence of different scavengers are performed (Fig. 10). For this aim, 1mM of isopropyl alcohol (IPA) (as ˙OH scavenger) and 1mM benzoquinone (BQ) (as O2˙− scavenger) is added to the MB dye. For the bare TiO_2_ case, addition of both of these scavengers have influenced MB degradation while for BQ contained solution MB degradation is more strongly quenched which means that O2˙− are the dominant species in this case. This dominancy is even more pronounced when we move to 1 cycle ZnO coated sample where photocatalytic degradation efficiency of MB dye is decreased from 98% to 31% in 270 min of irradiation under UV light. For the thick ZnO shell layer, it was shown that addition of none of these scavengers has effected MB dye degradation considerably. Revisiting these results, we can conclude that for thin ZnO shell layer, superoxide anion radical is the major oxidative species responsible for the photooxidative conversion of MB which is in agreement with our hypothesis.

## Conclusion

In summary, effectiveness of ultrathin ZnO shell layer on PCA of TiO_2_ NWs has been investigated. It was revealed that just a single layer of ZnO coating can significantly strengthen PCA of the cell for photodegradation of the organic pollutant (MB) under UV irradiation. Stemming from synergistic effects, the TiO_2_ sample coated with ZnO of a proper thickness (<3 ALD cycles) offers several advantages over the bare TiO_2_ sample including less density of surface traps, more efficient charge separation, smaller effective band gap, and better transport capability due to minimized diffusion path for minority carriers inside the shell layer. This paper demonstrates a systematic design and analysis methodology to achieve high photocatalytic activity. In our belief, the obtained results are significant not only for photocatalysis community but also it opens a new approach in heterostructures design for a wide range of applications such as photoelectrochemical water splitting and metal oxide based photovoltaic cells where higher short circuit currents can be attained as a result of reduction in recombination rates and enhancement in the absorption of the cell.

## Additional Information

**How to cite this article**: Ghobadi, A. *et al*. A Heterojunction Design of Single Layer Hole Tunneling ZnO Passivation Wrapping around TiO_2_ Nanowires for Superior Photocatalytic Performance. *Sci. Rep.* **6**, 30587; doi: 10.1038/srep30587 (2016).

## Supplementary Material

Supplementary Information
