# Supplementary material for: A Heterojunction Design of Single Layer Hole Tunneling ZnO Passivation Wrapping around TiO2Nanowires for Superior Photocatalytic Performance
Source: Sci Rep. 2016 Jul 28;6:30587. doi: 10.1038/srep30587 (PMC4964577; doi:10.1038/srep30587)
Supplement: Supplementary Information [file srep30587-s1.pdf]

Supporting Information

A Heterojunction Design of Single Layer Hole Tunneling ZnO Passivation Wrapping around TiO_2 Nanowires for Superior Photocatalytic Performance

*Amir Ghobadi[ǂ], Turkan Gamze Ulusoy[ǂ], Ruslan Garifullin, Mustafa O. Guler, Ali Kemal Okyay**

[*] Prof. A. K. OKYAY, T. G. Ulusoy, R .Garifullin, M. O. Guler

UNAM – National Nanotechnology Research Center, Institute of Materials Science and Nanotechnology, Bilkent University, 06800 Ankara, Turkey

E-mail: aokyay@ee.bilkent.edu.tr

Prof. A. K. OKYAY, A. Ghobadi

Department of Electrical and Electronics Engineering, Bilkent University, 06800 Ankara, Turkey

[ǂ]These authors contributed equally to this work.

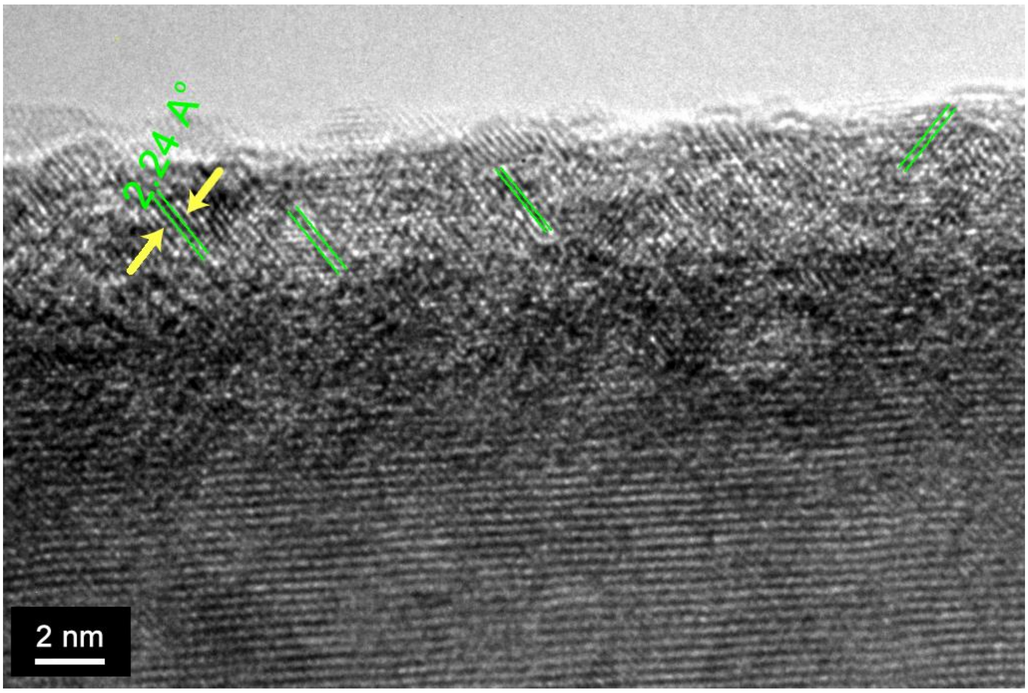

Figure S1. XPS spectra of (a) Ti2p and (b) Zn2p for the bare and coated heterostructures.

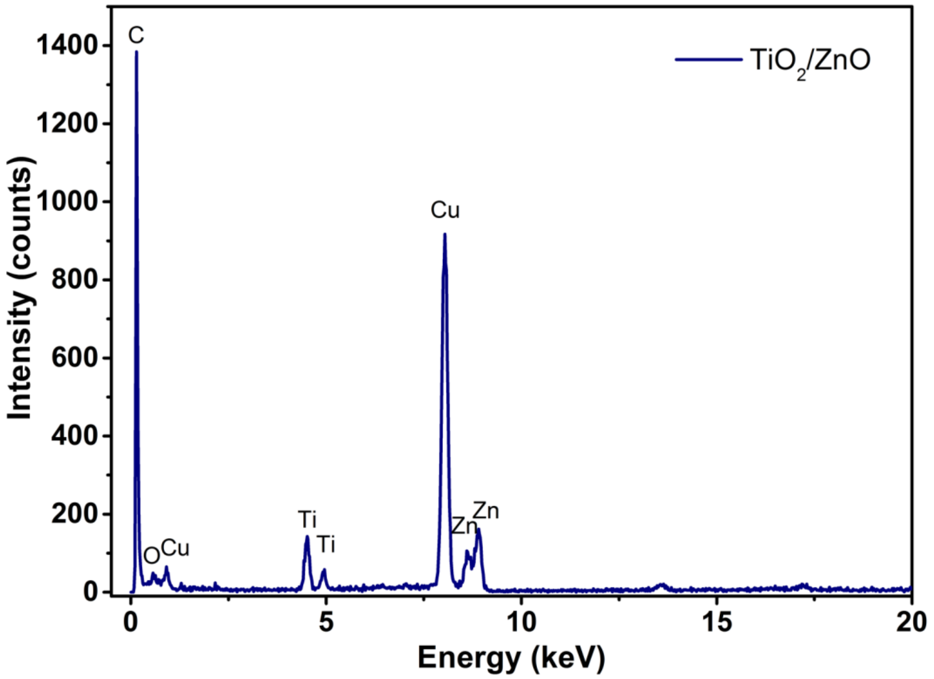

Figure S2. TEM EDS spectrum of the TiO_2-ZnO sample. Copper is from the form TEM grid.

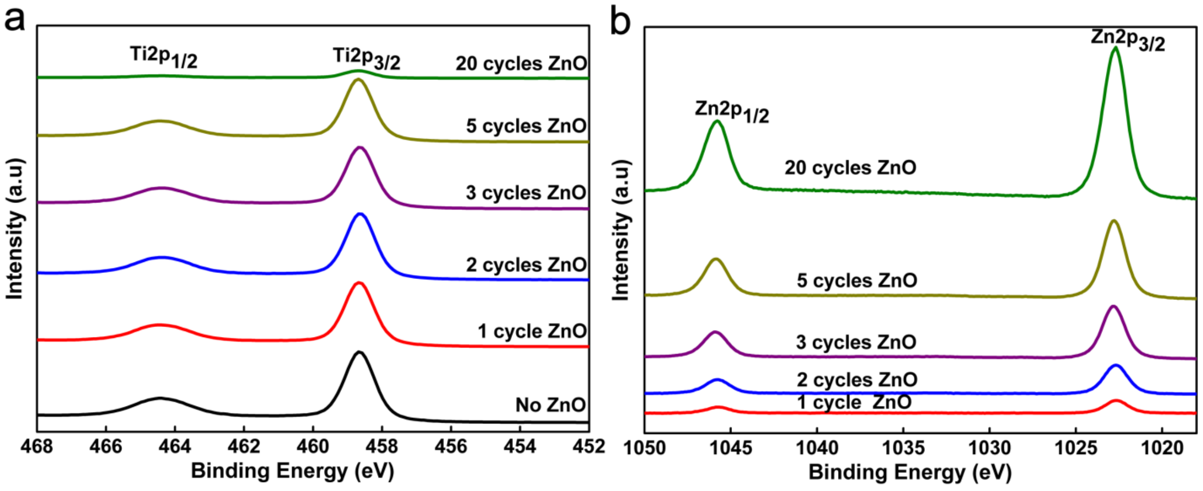

Figure S3. XPS spectra of (a) Ti2p and (b) Zn2p for the bare and coated heterostructures.

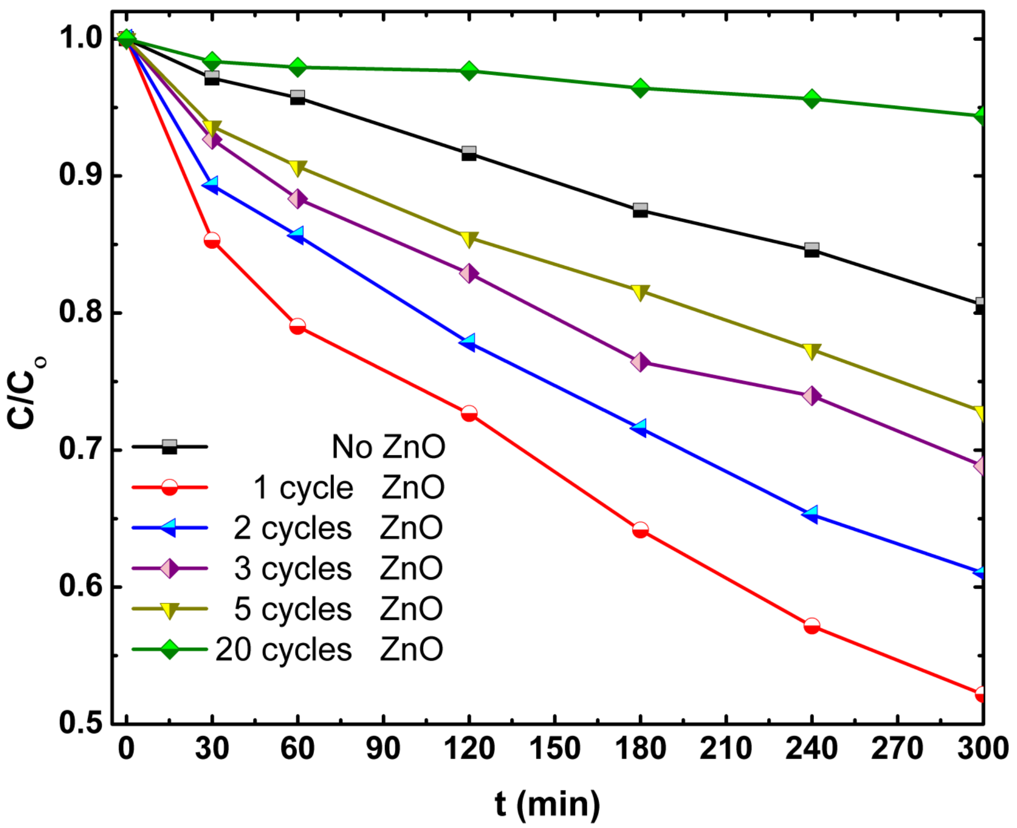

Figure S4. PCA of MB aqueous solution in the presence of different ZnO cycles and no catalyst cases (co=68.4 µM) as a function of the irradiation time.

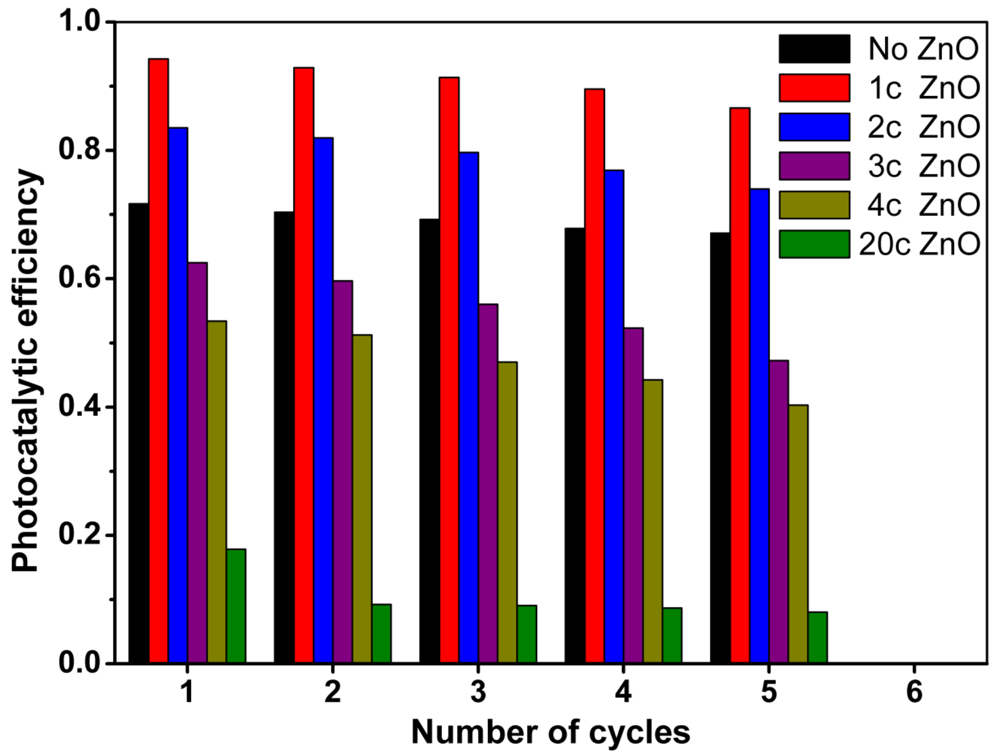

Figure S5. Recyclability test for bare and ZnO coated samples.
